# Back in Circulation: A Review of the Implementation of Thoracoabdominal Normothermic Regional Perfusion in Donation After Circulatory Death in Lung Transplantation

**DOI:** 10.3389/ti.2026.15272

**Published:** 2026-04-28

**Authors:** Anna Niroomand, Stephanie Chang, Sandra Lindstedt

**Affiliations:** 1 Department of Cardiothoracic Surgery, New York University Langone Health, New York, NY, United States; 2 Department of Cardiothoracic Surgery and Transplantation, Skåne University Hospital, Lund, Sweden

**Keywords:** DCD, donation after circulatory death, lung transplantation, TA-NRP, thoracoabdominal normothermic regional perfusion

## Abstract

In the face of a growing mismatch between candidates awaiting transplantation and the supply of conventional donor organs, attention has shifted toward novel methods to increase the donor pool, including the use of donation after circulatory death (DCD) and the refinement of procurement techniques that safeguard graft quality. Thoracoabdominal normothermic regional perfusion (TA-NRP) has emerged as a new strategy, leveraging extracorporeal support to curtail warm-ischemic injury while permitting *in situ* functional assessment. This review covers the rationale behind the use of TA-NRP, while outlining its use during procurement and the current body of evidence gathered on it implementation in lung transplantation specifically.

## Introduction and Overview of TA-NRP Methods and Implementation

In an era in which the need for transplant heavily outweighs the availability of suitable donors, the field of transplantation has turned to novel methods to increase the donor pool. Tactics to do so focus on increasing the quantity of donors and broadening the range of organs considered suitable, such as the utilization of extended criteria lungs. Aside from improving donor management and liberalizing donor criteria such as age or smoking history, the inclusion of donation after circulatory death (DCD) has gained considerable traction in recent years. While historically cadaveric lungs have come from donation after brain death (DBD), the use of DCD lungs has started to rise. These lungs were previously excluded due to concern over quality, however numerous studies have shown no significant difference in survival with DCD lungs. For donors where direct implant of the DCD allograft may be concerning for the transplant team, methods like normothermic regional perfusion (NRP) and *ex vivo* lung perfusion (EVLP) have enabled the safe evaluation and effective use of DCD lungs. Also known as non-heart-beating donors or as donation after circulatory determination of death, DCD is classified by the Maastricht classification system which differentiates between “uncontrolled” donors for whom cardiac arrest was unexpected and in which resuscitation was unsuccessful and “controlled” donors where care is withdrawn resulting in cardiac arrest [1, 2]. There are differences in the geographic distribution of DCD vs. DBD cases globally, with a higher prevalence of DCD use in Australia and Europe compared to the United States, though rates of utilization in the US are steadily increasing [3]. In controlled DCD, from which most organs are procured, life-sustaining support is withdrawn leading to a period of warm ischemia during which organs are not perfused but not yet cooled. The implementation of supportive systems following procurement, i.e., NRP, may mitigate the effects of warm ischemia, which is thought to be particularly damaging to the organs. This review will focus on thoracoabdominal NRP (TA-NRP) and its use in donor organ procurement, with a highlight on how its implementation has impacted lung transplantation.

### Procurement With TA-NRP

When procuring organs from the donor, organs can be recovered via the traditional direct procurement with cold flush and storage or alternatively by NRP whereby an extracorporeal membrane oxygenation (ECMO) circuit or another extracorporeal circuit like cardiopulmonary bypass (CPB) perfuses the organs. The use of a controlled reperfusion strategy both limits warm ischemic time and simultaneously allows for *in situ* evaluation of graft function [4–6]. The primary motivation behind the development of TA-NRP was the desire to expand heart transplantation to DCD donors, a population previously excluded due to irreversible myocardial warm ischemia. Conventional abdominal NRP maintained perfusion to abdominal organs but excluded the thoracic cavity, precluding safe cardiac recovery. By extending regional perfusion to include both thoracic and abdominal organs while isolating cerebral circulation, TA-NRP allowed for physiologic reperfusion, assessment, and recovery of the DCD heart.

Early use of NRP dates back to the 1980s and 1990s when Spanish groups performed high numbers of uncontrolled DCD procurement in liver transplantation. Unlike in other regions, the majority of DCD livers grew to be recovered with NRP and showed benefits in post-transplant outcomes such as biliary complications and graft loss compared to standard rapid recovery [7]. The use of abdominal NRP became routine in Spain and the UK, and early experiences at the Royal Papworth Hospital and within other UK centers pushed forward the development of TA-NRP [8, 9]. Adoption across the US has followed, with early experiences documented at Colorado, NYU and Vanderbilt accompanied by a growing popularity across centers in recent years [10].

**FIGURE 1:**
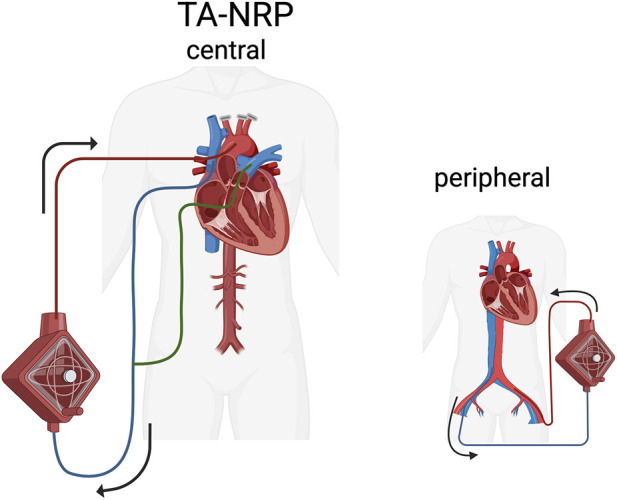
Configuration of thoracoabdominal normothermic regional perfusion (TA-NRP). Central (left) TA-NRP is accomplished with arterial inflow (red) via cannulation of the ascending aorta after ligation or clamping of the head vessels and venous drainage (blue) along with a pulmonary artery or left ventricular vent (green). Alternatively, peripheral TA-NRP (right) can also be performed through cannulation of the femoral vessels.

From a technical standpoint, TA-NRP can be initiated via central or peripheral cannulation (Figure 1). The femoral artery and vein are most commonly accessed for peripheral cannulation. This approach has the primary advantage of being able to cannulate prior to the procurement sternotomy. Central cannulation is most commonly initiated via the ascending aorta and through the right atrium, which is considered the standard cannula placement [11]. While there is no one codified technical approach to TA-NRP, a consensus statement on technical standards was published in August of 2024 drafted by a working group made of members from several surgical societies [11]. Standard of care as prescribed by that group stipulates that cerebral blood flow should be prevented before systemic perfusion starts. Cerebral reperfusion is typically impeded by the placement of clamps or ligation of all brachiocephalic vessels. Perfusion can be established with either an ECMO or CPB circuit, with preference depending on experience and availability. At its core, any circuit that is used will need to have a reservoir, oxygenator, pump, tubing and cannulas. It has however been noted that a CPB circuit does have some advantages including being able to unload the heart through venting, improving myocardial reperfusion, and the ability to correct derangements like hyperkalemia and metabolic acidosis which can occur with circulatory death [12]. Cardiac venting can be accomplished with a pulmonary artery vent which also facilitates lung procurement or with a left ventricular or a left atrial vent [11]. Many centers using ECMO for TA-NRP have modified the ECMO circuit to include a reservoir to allow for cardiac venting. Weaning off bypass/ECMO can be done with assessment of the graft’s function under a more normal physiologic setting to come to a better idea of if the heart can be accepted for transplant [12]. Regardless of whether CPB or ECMO is used, there are several critical factors that should be considered for every TA-NRP case. Importantly for the lung, the ventilatory parameters should be protective and include recruitment maneuvers to prevent perioperative lung injury. The length of time on TA-NRP is not yet standardized and there is no established ideal duration. If only thoracic organs are being procured, the duration of NRP should be limited to less than 60 min [11]. Organs can and should be assessed while off TA-NRP, particularly to evaluate the native cardiac function and can include pulmonary vein gas samples for donor lung evaluation. TA-NRP concludes with placement of the aortic cross-clamp, and start of the cold preservation time.

The risks of initiating TA-NRP are tied to the speed with which the chest should be entered, which could increase the likelihood of injury to major vessels like the inferior vena cava. Injury to the aorta could lead to a hematoma or dissection during cannulation, and the speed at which TA-NRP is initiated could lead to laceration of the right ventricle or lungs during the sternotomy and pericardiotomy [13]. There is also a risk of coronary air embolism with rapid cannulation which could lead to predonation arrhythmia [13]. These risks are mitigated by experience, which remains perhaps the biggest barrier to the use of TA-NRP along with the extent of resources it requires. As Moazami et al noted, the combined efforts of experienced cardiac anesthesiologists, transplant cardiologists, perfusionists, operating room personnel, and transplant surgeons are all needed to ensure the best chance at successful resuscitation of organs on bypass and for final acceptance [12]. As the recent consensus document notes, the surgeon performing TA-NRP should be experienced given the rapidity of cannulation and the need to initiate regional perfusion in a timely fashion. They suggest a credentialing process that includes an operative log of at least five cases for those with previous formal training or proctored recoveries for those without formal training. As the utilization of DCD organs becomes more commonplace, there are greater opportunities for providers and hospital systems to become familiar with the requirements and implementation of TA-NRP, which will eventually reduce impediments to its initiation.

## Use of TA-NRP in Lung Transplantation

TA-NRP can facilitate the procurement of several organs, with a prominent focus on cardiac and pulmonary harvest. For the lungs, the benefit is to quickly and efficiently re-initiate perfusion, which should mitigate effects of warm ischemia [14]. In the Colorado experience, the right atrium and distal innominate artery are cannulated, with a pulmonary artery vent placed at the level of arterial transection to allow for heart and lung splitting [15]. Through their technique, they report in a retrospective review that the median time from asystole to reperfusion was 9.5 min (IQR 8.75–12.5 min) with a total perfusion time of 86.5 min (IQR 79.75–92.5 min) [13]. From this cohort, they experienced only one instance of primary graft dysfunction (PGD) grade 2 in a single transplant recipient compared to 7 of 8 patients who had PGD grade 1 or less. Additionally, the median post-operative PaO_2_/FiO_2_ (PF) ratio increased over the course of post-operative days 0–2 from a starting point of 280.5 (236–255.5) to a peak of 370 (316.0–436.0). In another case study series out of the Leuven group in Belgium, five TA-NRP and one abdominal-NRP (A-NRP) cases were performed over the course of a year [16]. From this, two resulted in double lung transplants, though in contrast five hearts were accepted and transplanted. Of these two, one developed PGD grade 3 on post-operative day 2 and the other PGD grade 2 on post-operative day 3. Lungs which were not accepted after TA-NRP were rejected due to medical reasons.

In the NYU protocol, following cannulation and reperfusion, the patient is reintubated, bronchoscopy is performed, a left atrial vent is placed and after 30 minutes, the donor is weaned off support with an arterial blood gas drawn. NRP is then resumed until the aortic cross-clamp is ready to be applied and was run using either CPB or an ECMO circuit with a reservoir. With this, after a series of 8 lung transplants and 3 heart-lung procurements on TA-NRP, 100% of organs were utilized and there were no instances of grade 3 PGD at 72 h [17]. Compared to DBD donors, there were no significant differences in the PF ratios or short or long-term outcomes. From the Spanish experience which combined the results from several centers in the country, TA-NRP was performed in 28 lung cases and directly compared to 255 abdominal NRP (A-NRP) [18]. The TA-NRP group had no cases of PGD grade 3 at 72 h and had overall significantly lower rates of any grade of PGD compared to A-NRP. There were no differences in the rates or duration of mechanical ventilation, intensive care unit and hospital stays or in survival or readmission. In another retrospective analysis of transplanted lungs after TA-NRP from Vienna, there were no differences in rates of PGD or post-operative mortality when comparing concomitant heart-lung transplant to lung transplant alone [19]. Similarly, an analysis of the Scientific Registry of Transplant Recipients showed that among cardiac DCD donors, lung usage was similar between TA-NRP and direct procurement cases [20]. These case studies highlight the importance of monitoring post-operative PGD as well as organ utilization rates when trying to understand the impact of initiating TA-NRP as routine practice. The process of TA-NRP does not immediately appear to be correlated with high rates of severe PGD, however, the published papers are case series with limited numbers of implanted grafts. As experience with TA-NRP grows, any apparent relationship should become more evident. Experience will only grow if TA-NRP is deemed a useful tool in recovering organs, which appears to be the case given the utilization rates recorded across these case series. While utilization rate ranges from 64% in the 41 lungs transplanted from 70 lungs in the Spanish experience to the use of 100% in the NYU case series, TA-NRP appears to be a viable method of procurement to increase the donor pool.

Larger registry analyses have tried to find overarching relationships across comparison groups in multicenter data, finding suitable results within cases of TA-NRP pointing to no significant differences when compared to other forms of procurement. Across a query of the United Network for Organ Sharing (UNOS) database, of 627 DCD cases aimed at heart procurement, there were utilization rates of 14.9% of lungs in NRP cases compared to 13.8% in direct procurement [21]. Furthermore, there were no differences amongst in-hospital outcomes or short-term survival amongst the recipients in either group. Another UNOS registry data review from the same year looked at 434 DCD transplants, 17 of which were recovered by this classification with TA-NRP and demonstrated a lower likelihood of ventilation greater than 48 h and a non-statistically significant trend towards shorter hospital stays. Of 146 total TA-NRP donors in the pool the low number of 17 transplants equates to an 11.6% utilization rate, which is far lower than the 75.3% for cardiac transplant or 9.8% for kidney transplants [22]. In the STAR-OPTN dataset, 987 DCD lungs were analyzed, of which 92 underwent NRP without distinguishing between TA-NRP or A-NRP with no changes in short-term survival, PGD grade 3 or length of stay either. An important caveat to all three studies is that the TA-NRP groups consist of donors who are inferred to have gone through this type of procurement [23]. Each study relied on the time from asystole to cross clamp falling above a certain threshold of minutes to infer which were TA-NRP cases, which confers a degree of uncertainty as to the true status of each donor.

Use of TA-NRP has been criticized by some who cite concerns that its use can lead to pulmonary edema. Recommendations to minimize this complication including both donor management and technique. The Colorado experience notes that suitable donors are those with adequate urine output or on diuresis (with a suggestion of furosemide 20–40 mg every 8 h) to combat resulting pulmonary edema from both TA-NRP and cold storage [13]. They also for this reason limit their TA-NRP runs to 45–60 min in length, while considering extensions to 90–120 min if abdominal procurement requires more time. Other considerations in surgical technique include the placement of early pulmonary arterial venting and as the consensus document notes, drainage of donor blood after placing the venous cannula to reduce intracardiac pressures and combat venous congestion before TA-NRP perfusion starts [11]. The placement of the vent, while considered optional in the circuit for some, is noted by others to play an important role in reducing hydrostatic pressure [13]. One preclinical study has so far attempted to investigate the effects of TA-NRP on procurement through a porcine model by comparing TA-NRP to direct procurement via a post-procurement run on EVLP [24]. While the mean PA pressure increased during TA-NRP, the edema evaluated during EVLP and the lung injury scoring of the histopathology were comparable between TA-NRP and direct procurement groups.

## Evaluation of TA-NRP With EVLP

EVLP on its own has been discussed as a post-procurement method of evaluating and potentially rehabilitating donor organs both in DBD and DCD cases [25, 26]. Within the context of TA-NRP, EVLP has been proposed as an adjunctive method to further facilitate the evaluation of donor allografts and as a platform to deliver therapies [27]. In an abstract and subsequent article from Vanderbilt, a series of DCD lungs were put on EVLP at centralized centers and then categorized based on whether they were procured with TA-NRP, A-NRP or directly [28, 29]. The conversion rate following EVLP was found to be higher in TA-NRP compared to A-NRP but comparable to direct procurement. Across all of these groups, the need for post-operative ECMO was low at either 0 or 2 cases, however the length of hospital stay was significantly higher in both NRP groups. Other uses of EVLP with TA-NRP has been from case reports, including a study of a DCD recovery with TA-NRP run on EVLP for transport instead of cold storage. There was no finding of PGD following transplant and the authors concluded that the combination of TA-NRP with mobile EVLP served to reduce logistic challenges given the distance between the donor and the recipient [30]. These publications serve as limited proof that EVLP after TA-NRP can be both feasible and clinically useful, however, highlight a need for greater study of any synergistic effects.

## Conclusion

The current literature regarding TA-NRP suggests that it is a useful tool before the procurement of DCD organs. TA-NRP allows for transplant groups to leverage an intraoperative opportunity to mitigate the effects of warm ischemia while also evaluating organs *in situ*. Several case series from various groups internationally have both demonstrated the feasibility of TA-NRP as well as set the stage for standardization of the technique. Registry level analyses have also shown the use of TA-NRP leads to results equivalent to those of DBD donors in experienced centers. As Van Raemdonck et al have noted, however, a key tenet to maintaining good outcomes is good communication between teams involved in organ retrieval [31]. Additionally, presently, there is only a limited amount of data about TA-NRP and the controversial ethics surrounding TA-NRP demands the collection and analysis of more in-depth evidence to support its implementation. Registries should be modified to note when a donor case has proceeded with TA-NRP so that future studies do not infer the status from time intervals. As experience grows, single and multicenter retrospective analyses can create a clearer picture of clinical outcomes. These data sets will create the ability for centers to also further refine donor management, which has already demonstrated the need for certain practices like diuresis, venting during perfusion, and creating guidelines around the duration of TA-NRP. There may be opportunities to pair TA-NRP with other interventions such as EVLP to help facilitate logistic and clinical concerns. Prospective and adequately powered trials will always be necessary to validate long-term outcomes and understand the impact that these interventions have on rejection and survival metrics. Nevertheless, TA-NRP presents as a rapidly growing technique that certainly has its merits in organ procurement, with growing evidence demonstrating its utility in lung transplantation.
